# Teaching Musculoskeletal Module using dissection videos: feedback from medical students

**DOI:** 10.1186/s12909-021-03036-5

**Published:** 2021-12-07

**Authors:** Ayman G. Mustafa, Nour R. Taha, Sami Zaqout, Mohammed Seed Ahmed

**Affiliations:** 1 Department of Basic Medical Sciences, College of Medicine, QU Health, Qatar University, Doha, Qatar; 2 Biomedical and Pharmaceutical Research Unit, QU Health, Qatar University, Doha, Qatar; 3 Department of Anatomy, Faculty of Medicine, Jordan University of Science and Technology, Irbid, Jordan

**Keywords:** Anatomy, Dissection videos, Medical education, Medical students

## Abstract

**Background and Aims:**

Over the last two decades many medical schools have been exploring alternatives to hands-on cadaver dissection in teaching anatomy. This study aimed at reporting medical students’ feedback on using dissection videos in teaching anatomy of the musculoskeletal system.

**Methods:**

Dissection videos were used to teach the anatomy of the musculoskeletal system for third year medical students. At the end of the module, feedbacks from medical students were reported using a questionnaire designed for this purpose. Statistically valid responses were considered for 284 students.

**Results:**

Around 60% of the students enjoyed learning anatomy by watching dissection videos but the majority - mostly non-Jordanian - thought that the duration of the videos should be shorter. 83% (236/284)of the students enjoyed the presence of an instructor to guide them through the video and 85% (241/284) wanted to discuss the content with the instructor after watching. Most of the students liked to have access to the videos at any time in an open lab policy. Only 23% (66/284) of the students - mostly Jordanian – were willing to completely replace cadaveric prosections with dissection videos. Most of the students found that dissection videos helped them to understand anatomy lectures in a better way and in memorizing anatomical details. A significantly higher percentage of Jordanian students preferred watching dissection videos at home and preferred dissection videos to replace traditional anatomy lab sessions.

**Conclusions:**

In the light of our present findings, using dissection videos as a teaching method of anatomy was well received by students. However, it seemed that the students wanted dissection videos to be integrated with using cadaveric prosections rather than replacing them.

## Background

Anatomical courses have an integral role in building the solid preclinical knowledge perused by any medical school. Practicing physicians should have a thorough understanding of human anatomy in order to perform physical examination, symptom interpretation, surgical procedures, and other medical interventions [1–3]. As such, medical schools bear the burden of providing quality anatomy education that allows students to acquire the anatomical knowledge they will need in their medical practice. The science of anatomy in itself is considered challenging due to the large quantities of information, new terminologies, and complex material students are expected to learn [4]. Cadaveric dissection has long since been the cornerstone of anatomy education [5] and while beneficial, dissection does have its drawbacks. This raises the debate as to whether using cadaveric dissection as an educational tool is necessary or if it should be replaced with non-cadaveric approaches that take advantage of the available advanced technologies [6]. Taking a thorough look at this debate, one can see that both sides have merit. On one hand, cadavers are excellent for teaching the anatomy of large organs, showing a range of normal anatomical variation, and delivering clinically oriented anatomical information [7–9]. Furthermore, they enable students to understand the body structures in three-dimensional form through sensory and tactile perception [10]. Accordingly, many researchers and educators believe that maintaining “hands-on” cadaver dissection is vital and indispensable for anatomy education [11, 12]. On the other hand, dissection may be considered ineffective in learning some anatomical topics such as surface anatomy, the anatomy of small indistinct organs, nerves, vessels and lymphatics [7]. Moreover, to some students, the dissecting room is a cause of stress and anxiety as they are not familiar with the emotional responses that arise when encountering a deceased human being [13–15]. An additional obstacle for students may be learning dissection techniques that require guidance and are exceptionally time consuming, which does not allow them to actually locate the structures they are supposed to be finding [10]. For these reasons and many more, some researchers, educators, and even students believe that using dissection alone is insufficient to learn anatomy, and that incorporating other educational tools is advantageous, or even required [7, 16–18]. It is interesting to note that the use of additional educational resources is not exactly a brand new concept, since “motion picture” tools have been utilized since as far back as the 1970 s in the form of VHS tapes that then progressed to CD Roms and eventually the multiple video tools that are available today [19]. While the debate is still ongoing, many medical schools have opted to put dissection on hold due to the increased number of students, the shortage of time, cadavers, and qualified staff. In fact, the availability of other tools including plastic models and plastinated specimens offer suitable alternatives [20].

The past few years have witnessed a quantum leap in medical education. In the field of anatomy, there is an observed trend toward using computer-based and multimedia-assisted educational tools such as animations, three-dimensional models, virtual microscopy and videos. Anatomy-related videos may include animations, illustrations, clinical anatomy cases, and dissection videos. However, the actual usage of such videos, as well as the academic impact of student use of these videos, is largely unknown [10]. Consequently, the effect of implementing such tools in teaching anatomy has been the target of several investigations [18, 20–26]. The positive value of using anatomy-related videos was reported by many studies [16, 26–29]. Dissection videos can be used to demonstrate how to complete the dissection of a certain body part [10] and may even act as a replacement for dissection instructors using atlases [30] thus saving a magnitude of time. Considering the multiple reasons as to why real time dissection is not feasible in many medical schools, pedagogically using dissection videos seems to be a track worthy of much more investigation.

### Study goals

In this work, the potential usefulness of using dissection videos in teaching anatomy of the musculoskeletal system as perceived by medical students studying at Jordan University for Science and Technology (JUST) was explored. This study investigated students’ attitude toward incorporating dissection videos as a teaching tool into the musculoskeletal module offered at JUST. This study aimed first to reveal whether students were positive or negative toward using dissection videos. For students with positive attitude, the study also aimed to report whether they would like dissection videos to be integrated in current anatomy labs, or entirely replace current anatomy labs. To the best of our knowledge, this is the first study to explore using dissection videos as an anatomy-teaching tool in Jordanian medical schools. The outcome of this work is expected to shed more light on the potential value of dissection videos as an alternative or an additional tool to models and cadaveric prosections.

## Materials and methods

This work was conducted on third year medical students who were, as per their curriculum, enrolled in the musculoskeletal module offered by faculty of medicine at JUST. To attain the purpose of the study, 40-45-minute Acland’s dissection videos were used as an alternative to regular lab sessions. The videos were played by an anatomy instructor. The instructor provided comments on the videos, guided the students, and answered their questions. To explore the students’ attitude toward using this method, an anonymous paper-based questionnaire was designed and administered to the students at the end of the module. The study was approved by the institutional review board (IRB) at JUST (2019-147).

### Educational context

In Jordan, Students enroll into the medical school after finishing high school. The medical curriculum at Just is a six-year curriculum consisting of three phases. Phase I of the medical curriculum is covered during the first year and half of medical school. During this phase, which consist of life sciences and general courses of basic medical sciences, one general course of anatomy is offered to students. This course is taught by didactic lectures and practical labs using models, plastinated specimens, and prosections.

Phase II of the medical curriculum is covered in the second semester of year 2 and both first and second semesters of year three. In this phase, basic medical sciences are taught through system-based integrated modules. In each module, the relevant anatomy is taught by lectures and practical labs. The module with the largest proportion of anatomy is the musculoskeletal module offered to third year medical students. The anatomy of the musculoskeletal system was taught by didactic theoretical lectures as well as weekly two-hour practical sessions. Anatomy labs were a self-learning environment for the students who learnt anatomy utilizing models, plastinated specimens, and cadaveric prosections. In addition to that, problem-based sessions were used to discuss some clinical cases emphasizing the clinical correlation between the anatomical information delivered through the module and clinical practice.

Phase III of the medical curriculum consists of clinical training in years 4, 5, and 6. No courses in anatomy are offered during this phase.

### Questionnaire design

The 1st two questions identified each participant’s gender and the Jordanian from non-Jordanian students. The rest of the questionnaire consisted of 14 (agree/disagree) statements. These statements were designed to inquire about the students’ satisfaction with using dissection videos and with the way these videos were delivered. The statements also explored the students’ perceived usefulness of dissection videos in understanding, memorization, and exam performance. Lastly, the participants were asked if they prefer using dissection videos as a substitute for regular lab sessions and lectures, and if they recommend using this method for other students in other courses.

### Data collection

302 copies of the questionnaire were distributed on the 302 students registered in the musculoskeletal system. 284 (94%) completely answered questionnaires were obtained. The non-responders were 18 (6%). These represent questionnaires with unattepmted questions or without reporting gender and/or nationality, which were excluded from this study. In addition to the background Data, 14 items were developed from the questionnaire and subjected to further processing.

### Data processing and analysis

The statistical analysis was performed using the Statistical Package for Social Sciences software, version 28 (SPSS, Inc., Chicago, IL). The collected data were numerically coded for statistical analysis. A positive answer was given code one, and a negative answer was coded zero. To reveal any statistical differences related to gender or nationality, Pearson’s Chi-square test was used. Differences were considered significant whenever the P value was less than 0.05 (P < 0.05). A power calculation was conducted using Alpha level of 0.05 and a conservative proportion for the main outcome variables for the provided sample size. The calculations revealed a power of more than 80%.

## Results

### Background of the participants

All the participants were third-year medical students who voluntarily filled out the questionnaire at the end of the musculoskeletal module. Their demographic data are illustrated in Table 1.

**Table 1:** Background information and Characteristics of the study sample (n = 284)

	n (%)
**Nationality**	
Jordanians	158 (56%)
Non-Jordanians	126 (44%)
**Gender**	
Males	167 (59%)
Females	117 (41 %)
**Level of Study**	
Third Year	284 (100.0)

### Dissection videos experience

The results showed that 60% (169/284) of the students enjoyed learning anatomy by watching dissection videos. On the other hand, 79% (224/284) reported that watching dissection videos in the lab was boring, and 89% (253/284) think that the duration of the videos should be shorter.

83% (236/284) of the students liked the presence of an instructor to guide them through the video. Moreover, 85% (241/283) preferred to discuss the content with the instructor after watching. The majority of the students favored having access to the videos in an open lab policy. This also coincided with the fact that 80% (227/284) wanted to watch dissection videos at their own convenience at home. Even though the students were very positive about using dissection videos, they still wanted to use cadaveric prosections. Only 23% (67/284) of the students were willing to use dissection videos as a complete alternative to cadaveric prosections. Having said that, there remains a general positive trend toward dissection videos as 42% (120/284) preferred this method to replace traditional lab sessions, and 77% (219/284) recommended using dissection videos for other students in other courses, (Table 2).

**Table 2:** Percentages of positive and negative responses of all students.

Questionnaire Items	Response	
	% Positive (n)	% Negative (n)
**Part 1: Feedback on Dissection Videos Experience**		
I enjoyed learning anatomy by watching dissection videos.	60 (170)	40 (114)
Watching dissection videos in groups in the lab is boring.	79 (224)	21 (60)
I prefer having an instructor to guide me through the video.	83 (236)	17 (48)
I prefer watching dissection videos at my own convenience at home.	80 (227)	20 (57)
I prefer discussing the content of dissection videos with an instructor after watching.	85 (241)	15 (43)
The duration of dissection videos should be shorter.	89 (253)	11 (31)
I prefer having access to the dissection videos at any time (open lab policy).	98 (278)	2 (6)
I prefer learning anatomy by dissection videos rather than by cadaveric prosections.	23 (65)	77 (219)
I prefer dissection videos to replace traditional anatomy practical sessions.	42 (119)	58 (165)
I recommend using dissection videos for other students and other courses.	77 (219)	23 (65)
**Part 2: Perceived Usefulness of Dissection Videos**		
Dissection videos provided sufficient anatomical knowledge for medical students with no need for lectures.	35 (99)	65 (185)
Dissection videos helped me understand anatomy lectures in a better way.	85 (241)	15 (43)
Dissection videos helped me memorize anatomical details in a better way.	76 (216)	24 (68)
Dissection videos helped me increase my grades at anatomy exams.	60 (170)	40 (114)

### Perceived usefulness of dissection videos

85% (242/284) of the students reported that dissection videos helped them to understand anatomy lectures in a better way. In addition, 76% (215/284) reported that these videos were helpful in memorizing anatomical details. A percentage of 35 (100/284) found that dissection videos provide sufficient anatomical knowledge for medical students with no need for lectures. Moreover, 60% (169/284) reported that dissection videos helped them increase their grades in anatomy exams, (Table 2).

### Results in relation to gender

Statistical analysis of the responses revealed that significantly more male students were in favor of using dissection videos over traditional anatomy lab sessions (p < 0.05) (Table 3).

**Table 3:** Students’ responses in relation to gender. Pearson’s Chi-square test was used to compare percentages of positive responses between male and female students

Questionnaire Items	% Positive response		P value
	M	F	
**Part 1: Feedback on Dissection Videos Experience**			
I enjoyed learning anatomy by watching dissection videos.	62 (104/167)	56 (66/117)	0<.05
Watching dissection videos in groups in the lab is boring.	75 (125/167)	84 (98/117)	0<.05
I prefer having an instructor to guide me through the video.	82 (137/167)	85 (99/117)	0<.05
I prefer watching dissection videos at my own convenience at home.	77 (129/167)	84 (98/117)	0<.05
I prefer discussing the content of dissection videos with an instructor after watching.	86 (144/167)	83 (97/117)	0<.05
The duration of dissection videos should be shorter.	87 (145/167)	92 (108/117)	0<.05
I prefer having access to the dissection videos at any time (open lab policy).	96 (160/167)	99 (116/117)	0<.05
I prefer learning anatomy by dissection videos rather than by cadaveric prosections.	27 (45/167)	18 (21/117)	0<.05
I prefer dissection videos to replace traditional anatomy practical sessions.	50 (84/167)	31 (36/117)	<0.05
I recommend using dissection videos for other students and other courses.	77 (129/167)	77 (90/117)	0<.05
**Part 2: Perceived Usefulness of Dissection Videos**			
Dissection videos provided sufficient anatomical knowledge for medical students with no need for lectures.	38 (63/167)	31 (36/117)	0<.05
Dissection videos helped me understand anatomy lectures in a better way.	85 (142/167)	86 (101/117)	0<.05
Dissection videos helped me memorize anatomical details in a better way.	78 (130/167)	73 (85/117)	0<.05
Dissection videos helped me increase my grades at anatomy exams.	62 (104/167)	56 (66/117)	0<.05

M: male students

F: female students

### Results in relation to student’ background

Statistical analysis of students’ responses showed that compared to non-Jordanians, a significantly higher percentage of Jordanian students preferred watching dissection videos at home (p < 0.05), and preferred dissection videos to replace traditional anatomy lab sessions (p < 0.05). Moreover, a significantly higher percentage of Jordanian students reported that dissection videos helped them to understand anatomy lectures (p < 0.05), and to memorize anatomical details (p < 0.05). On the other hand, a significantly higher percentage of non-Jordanian students believed that the duration of the videos should be shorter (p < 0.05) (Table 4).

**Table 4:** Students’ responses in relation to nationality. Pearson’s Chi-square test was used to compare percentages of positive responses between Jordanian and Non-Jordanian students

Questionnaire Items	%Positive response		P value
	J	NJ	
**Part 1: Feedback on Dissection Videos Experience**			
I enjoyed learning anatomy by watching dissection videos.	63 (100/158)	55 (69/126)	0<.05
Watching dissection videos in groups in the lab is boring.	80 (126/158)	77 ( 97/126)	0<.05
I prefer having an instructor to guide me through the video.	80 (126/158)	87 (110/126)	0<.05
I prefer watching dissection videos in my own convenience at home.	85 (134/158)	73 (92/126)	0<.05
I prefer discussing the content of dissection videos with an instructor after watching.	85 (134/158)	84 (106/126)	0<.05
The duration of dissection videos should be shorter.	85 (134/158)	94 (118/126)	<0.05
I prefer having access to the dissection videos at any time. (open lab policy)	97 (153/158)	98 (123/126)	0<.05
Dissection videos provided sufficient anatomical knowledge for medical students with no need to lectures.	35 (55/158)	35 (44/126)	0<.05
I prefer learning anatomy by dissection videos rather than by cadaveric prosections.	19 (30/158)	29 (37/126)	0<.05
I prefer dissection videos to replace traditional anatomy practical sessions.	48 (76/158)	36 (45/126)	<0.05
I recommend using dissection videos for other students and other courses.	77 (122/158)	78 ( 98/126)	0<.05
**Part 2: Perceived Usefulness of Dissection Videos**			
Dissection videos helped me understand anatomy lectures in a better way.	92 (145/158)	77 (97/126)	<0.05
Dissection videos helped me memorize anatomical details in a better way.	82 (130/158)	68 (86/126)	<0.05
Dissection videos helped me increase my grades at anatomy exams.	60 (95/158)	59 (74/126)	0<.05

J: Jordanian students

NJ: Non-Jordanian students

## Discussion

Pedagogical usage of the advancing multimedia represents a quantum leap in medical education. More research nowadays is directed towards studying the effect of using images, animations, videos, and other visual tools in learning anatomy [6, 17, 18]. Indeed, this trend probably evolved to suit the needs and preferences of students, cope with modernized pedagogical tools, and compensate for the absence of traditional dissection in many medical schools [2]. The current advance in the production of high-quality dissection videos using typical human specimens raised the question whether such videos can be integrated beneficially in the medical curriculum as extraordinary educational tools during anatomy sessions. The answer to this question is of special interest for medical students since viewing the aforementioned videos is quite enjoyable for them in comparison to dissection room sessions.

In the present study, the collected data showed that 60% (169/284) of the students enjoyed learning anatomy by watching dissection videos. The rest of the students were not against dissection videos *per se* but rather most of them did not like watching the videos in structured lab sessions. This was evident as 78.9% (224/284) of the students reported that watching dissection videos in groups during lab sessions was boring to them. It seemed that students found dealing with plastinated models and cadaveric prosections in groups more interesting and interactive for a structured lab session. A very close percentage (80%) (227/284) preferred watching dissection videos at their own convenience at home. Thus, students appeared to enjoy watching dissection videos as a self-learning tool. The majority of the students (89%) (253/284) reported that the duration of the videos should be shorter. This comes in line with previous studies, which showed that short videos attracted learners’ attention much more effectively [31, 32]. One study focused specifically on the use of short videos in musculoskeletal anatomy. The investigators used self recorded videos, ranging from 14 s to 1 min 13 s, to identify muscles of the limbs and their relations by area. They reported that the shortness of the videos made them easy accessible to the students and that they could use them repeatedly [19]. Most students valued the presence of an instructor during (83%) (236/284) and after (85%) (241/284) watching the videos. his social presence is understandable since the learning process is enhanced by interaction and sharing ideas.

Regarding the perceived usefulness, most students reported that dissection videos helped them to understand anatomy lectures (85%) (242/284) and to memorize anatomical details (76%) (214/284). This was reflected by the slight increase in the number of students who experienced an increase in their anatomy exams’ grades (59.5%) (169/284). Similarly, a group of first year students at the University of Nebraska have improved practical lab test scores when they utilized dissection videos as a new study tool [30]. When considering the use of teaching videos and their effect on student grades, however, results have generally been variable. Some researchers have found their effect to be positive but in some cases the grades remained unchanged [10, 30]. Results from the present investigation also support the findings of previous studies, where watching such videos had multiple benefits. These included an enhancement in the learning process, retention of the perceived knowledge, and improved learning outcomes [33, 34]. The students at the University of Queens (Australia) gave feedback supporting the use of supplementary instructional videos in learning the anatomy of the upper limb. They reported an increased familiarity with the specimens and anatomical structures they would see in the practical class. The videos also helped them with class preparation and exam revision [4]. Only 35% of overall students believed that these videos could provide sufficient anatomical knowledge to completely replace traditional lectures. This could be a reflection of the fact that lectures are indispensable in higher education [35]. An Iranian cross sectional study evaluated the knowledge of 225 preclinical and clinical students on the methods of teaching anatomy and they too found that lectures were perceived to be the most effective instructional method [36].

The differences reported in some aspects of this study between Jordanian and non-Jordanian students can be ascribed to the difference in teaching methodology expectations between both groups. For instance, most non-Jordanian students were not of the opinion that dissection videos can completely change traditional anatomy lab sessions. This might be due to the fact that most international students come from countries where using hands-on anatomy lab is the first line teaching approach in medical school. On the other hand, Jordanian students might be inclined toward relying on dissection videos because they consider hands-on dissection of cadavers very stressful [37]. Clearly, there is huge cultural impact on learning anatomy [38, 39].

Analysis of the data in relation to gender revealed striking differences between female and male students in two closely related aspects. A lower percentage of females were interested in complete replacement of traditional anatomy practical sessions by dissection videos. Moreover, the percentage of females who wished to continue using cadaveric specimens was higher than males. A plausible explanation could be gender-related; spatial ability and mental rotation skills, which are central in learning anatomy, differ between females and males [40]. Indeed, the existence of gender-related variations among medical students regarding generic spatial ability and anatomy mental rotation tests has been documented in the literature [41, 42]. Therefore, female medical students might be relying on models and cadaveric prosections to augment their three-dimensional visualization of anatomical structures and enhance their mental rotation skills leading to a better memorable understanding.

### Limitations

Despite the potential virtues of dissection videos, they are not an interactive method of instruction. This is why many students did not want to use them in a structured lab format but rather at their own convenience. Moreover, the tactile and psychomotor components of learning were undermined by using dissection videos [43]. The importance of these skills explains why most students were not willing to stop using cadaveric prosections. Other limitations were related to the study design. The study only included one batch of third-year medical students and only involved one module of the medical curriculum. The study could be repeated to span multiple academic years thus including more batches of medical students as well as all the system-based modules in the curriculum.

## Conclusions

In the light of our present findings, integrating dissection videos into the medical curriculum was well received by medical students. Obviously, students were satisfied with this instructional method and believed it fostered their grasp of anatomical sciences. In particular, students were interested in using dissection videos on their own convenience at home. Moreover, most students wanted to continue using cadaveric prosections in addition to dissection videos. Finally, consideration should be taken to avoid the use of long videos in order to make them more enjoyable and attractive for the students.

## Data Availability

The datasets used and/or analyzed during the current study are available from the corresponding author on reasonable request.

## Abbreviations

JUST: Jordan University of Science and Technology
IRB: Institutional Review Board
F: Females
M: Males
J: Jordanian students
NJ: Non-Jordanian students
